# What Important Part Does Alcohol Play in the Economy of Man

**Published:** 1875-10

**Authors:** Charles P. King


What Important Part Does Alcohol Play in the Economy of Man?—*By Charles P. King, M.D.*— * * * *First.* What is alcohol? It is defined as being a product which results from the vinous fermentation in substances containing grape sugar. At a temperature of 80° Fahrenheit, the presence of a fermenting body converts the solution into carbonic acid and alcohol. Its preparation may be divided into three stages: The production of a fermented vinous liquor; the preparation from this of an ardent spirit by distillation; and lastly, rectification or purification. * *

What important part does alcohol play in the economy of man? To say that alcoholic liquors should be discarded entirely from use simply because they contain *poison*, is certainly no valid argument against a proper use of them; for we might use the same argument against nearly all of our ordinary articles of food, for they all contain more or less poisonous elements. There is poison in our common garden lettuce and in the hops with which we raise our bread. The oils contained in our table mustard and pepper, and in that healthy vegetable, the onion, are among the most destructive poisons with which the chemist is familiar. * * * After repeated experiments, the most reliable chemists and physiologists have shown that when alcohol is taken into the system, "in very small quantities," it disappears in the body. Neither in the breath nor perspiration, nor in any other secretion, can alcohol be found; but if taken in very large quantities it leaves the body again as alcohol. The amount of alcohol decomposed in the body corresponds exactly with the amount of oxygen which the body is able to furnish. * * * When used largely it impairs the appetite, and may almost entirely destroy it, but when taken in moderate quantities is assimilated and becomes food. Every physician of experience knows how almost indispensable this agent has become in the treatment of many of our low forms of fever; and life has been prolonged, nay, even saved, in many cases, by its administration, where ordinary food could not be administered. * * * Thus, within certain limits of dose, alcohol is transformed, like ordinary food, into the system, without producing any injurious effects; and, yielding useful force for the purpose of economy, must be considered as food in any philosophical sense of the word. And an important point to know, and one little understood, is that this food action is attended with no exciting or intoxicating influence; but the whole effect, like that of ordinary food, is seen in the maintenance or restoration, according to circumstances, of that balance or function called health. But, if taken in greater quantity than can be utilized as a force-yielding food, the excess of alcohol acts as a poison, producing a well-known train of perturbations of function. And—a point again generally misunderstood—all signs of departure from the natural condition in the drinker, from the first flushing of the face, brightening of the eye, and unnatural mental excitement, to the general paralysis of complete drunkenness, belong equally to the poisonous effects of alcohol; that is (for I wish to be understood as strongly insisting upon this point), even the early phases of alcoholic disturbance, which are often improperly called 'stimulating,' are part and parcel of the injuriously disturbing influences of overdoseage, and must be put in the same catagory with the more obviously poisonous effects of pronounced intoxication.

Alcohol has thus a two-fold action: First. It is capable, in proper dose, of being consumed and utilized as a force producer, in which case there is no visible disturbance of normal function. Such action cannot be distinguished by the drinker or physiologist from that of a quickly digestible fluid food, and is no more an "excitant" or "stimulation," followed by a "recoil" or "depression," than is the action of a bowl of hot soup or a glass of milk.

The second action is the poisonous influence of an excess of alcohol circulating in the blood, which makes itself sensible to the drinker by peculiar sensations and disturbances, and is not only followed by "depression," but is itself a form of depression; that is, a disturbance of balance—an unnatural perturbation of the normal working of the functions.

Every reader of these lines will at once ask: What then is the limit as to the quantity within which alcohol exerts only a food action, and beyond which it begins to poison by its excess? This question cannot be answered categorically; for it so happens that the "poison line," as it has been aptly called, is a shifting one. Even in health it varies according to age, sex, individual peculiarities and habits; and even in the same person, according to his physical condition for the time being—when fatigued by bodily or mental work; when suffering from an emotional agitation—as anxiety or fear; when worn by loss of sleep or blood, or by pain, amounts which ordinarily would flush the face, and somewhat confuse the mind, will be borne without producing the slightest symptoms of intoxication, the whole effect of the drink being expended in relieving the perplexing malaise, and restoring the system to its normal condition. And in more formal morbid states as in many diseases, the poison line often shifts to an alarming degree, so that what would in health produce even dangerous drunkenness, will be borne without causing the least intoxication, the whole of the alcohol being apparently utilized by the system for obtaining the life-saving energy which this fluid, from its swift absorption and ready chemical change in the blood, can so quickly yield.

Much opprobrium, of late years, has been heaped upon the medical profession on account of their indiscriminate use of alcoholic liquors in their prescriptions; and in some cases they may be justly deserving of censure. We would say but one word on that point, and that is this, that we, as physicians, should never lose sight of the fact that alcoholic stimulants are agents potent for evil as well as good. They should be administered with great care, and their use diminished or discontinued as soon as practicable. * * *

Physicians can not regard themselves as free to say they shall or shall not, under any circumstances use this, that, or the other remedy in the treatment of diseases. They are bound to use whatever remedies science and experience have taught them to be efficacious in the cure of disease. If the physician is called into a case where there is an extreme and perilous degree of prostration, and where a slower remedy would not answer, thus saving the patient's life, then that physician should have no remorse of conscience for having used alcoholic stimulants, although drunkenness might follow. The patient himself, not the physician, would be responsible for the bad results. From a long experience and observation, we believe that in health the use of stimulants is injurious—the person is better off if he abstains entirely from their use; but in some forms of disease, good results often accrue from the proper administration of them.

To prohibit stimulants entirely from our modern civilization would be as morally impossible as it would to prohibit the use of beef, or bread, or fruit. They are woven in, and have become a part and parcel of our modern society, and will probably continue to be so until that society shall perish from the face of the earth.

In conclusion we would say that this is our honest conviction that the temperance cause would be much stronger in our own country to-day, and the total abstinence principle commend itself more readily to the people, were all the facts concerning alcohol admitted, and an appeal made to the intelligent judgment and consideration of men on the basis of these facts. Nothing is ever gained by misrepresentation. If alcohol is, in all cases and in all quantities, a poison, let us know it and be confirmed in that fact. If, however, it is not a poison when properly used, let us be just as free to admit it, and leave the whole matter to the intelligent judgment of the people.

We are not of that number who believe in the indiscriminate use of alcoholic liquors. On the contrary, we have ever advocated that they should be used with great care and circumspection. They should be used and not abused. An All-wise Creator has given them to us for our good; and, if properly used, will subserve to the wants and happiness of man; but if abused, they will prove, like many other good things in this world, a curse rather than a blessing.—*Cincinnati Lancet and Observer.*

